# Oxidative Burst-Dependent NETosis Is Implicated in the Resolution of Necrosis-Associated Sterile Inflammation

**DOI:** 10.3389/fimmu.2016.00557

**Published:** 2016-12-01

**Authors:** Mona H. C. Biermann, Malgorzata J. Podolska, Jasmin Knopf, Christiane Reinwald, Daniela Weidner, Christian Maueröder, Jonas Hahn, Deborah Kienhöfer, Alexandre Barras, Rabah Boukherroub, Sabine Szunerits, Rostyslav Bilyy, Markus Hoffmann, Yi Zhao, Georg Schett, Martin Herrmann, Luis E. Munoz

**Affiliations:** ^1^Department of Internal Medicine 3 – Rheumatology and Immunology, Universitätsklinikum Erlangen, Friedrich-Alexander-University Erlangen-Nürnberg, Erlangen, Germany; ^2^UMR CNRS 8520, Institut d’Electronique de Microélectronique et de Nanotechnologie (IEMN), Université Lille 1, Villeneuve d’Ascq, France; ^3^Danylo Halytsky Lviv National Medical University, Lviv, Ukraine; ^4^Department of Rheumatology and Immunology, West China Hospital, Sichuan University, Chengdu, China

**Keywords:** necrosis, inflammation, nanodiamonds, NETosis, resolution, reactive oxygen species

## Abstract

Necrosis is associated with a profound inflammatory response. The regulation of necrosis-associated inflammation, particularly the mechanisms responsible for resolution of inflammation is incompletely characterized. Nanoparticles are known to induce plasma membrane damage and necrosis followed by sterile inflammation. We observed that injection of metabolically inert nanodiamonds resulted in paw edema in WT and Ncf1** mice. However, while inflammation quickly resolved in WT mice, it persisted over several weeks in Ncf1** mice indicating failure of resolution of inflammation. Mechanistically, NOX2-dependent reactive oxygen species (ROS) production and formation of neutrophil extracellular traps were essential for the resolution of necrosis-induced inflammation: hence, by evaluating the fate of the particles at the site of inflammation, we observed that Ncf1** mice deficient in NADPH-dependent ROS failed to generate granulation tissue therefore being unable to trap the nanodiamonds. These data suggest that NOX2-dependent NETosis is crucial for preventing the chronification of the inflammatory response to tissue necrosis by forming NETosis-dependent barriers between the necrotic and healthy surrounding tissue.

## Introduction

Sterile inflammation usually occurs as a reaction to tissue injury and cell death (1). Endogenous molecules released from necrotic cells (damage-associated molecular patterns, DAMPs) usually trigger and augment the inflammatory response to the insult and cell necrosis (2). For instance, the high-mobility group box 1 (HMGB1), a *bona fide* DAMP is released from necrotic cells (3) and triggers a neutrophil-mediated injury amplification loop that involves the receptor for advanced glycation end products (4). Neutrophils, the most abundant leukocytes in blood, are considered the initial line of defense, as they are the first cells recruited to sites of injury. Upon recruitment, neutrophils produce ROS, degranulate, and release pro-inflammatory cytokines to ensure the inactivation of the putative aggressor (5).

Importantly, neutrophils also release neutrophil extracellular traps (NETs) to entrap and kill microorganisms (6). This suicidal process is referred to as NET formation or NETosis (7). NETs are released in a coordinated series of events that involve chromatin decondensation and translocation of granular proteins. Upon neutrophil activation, the integrity of the granular membranes is lost. In consequence, neutrophil elastase (NE) and myeloperoxidase (MPO) translocate to the nucleus where they contribute to histone degradation and chromatin decondensation, respectively (8, 9). PAD4-mediated citrullination of histone H3 (citH3) has been shown to foster chromatin decondensation (10, 11). Reactive oxygen species (ROS) contribute to the release of NE and MPO from the granules and their translocation to the nucleus (8, 12) and probably at later stages to the disruption of the plasma membrane. Released DNA gets then decorated with NE, MPO, and citH3. Besides pathogens, other stimuli, such as cytokines (13), phorbol myristate acetate (PMA) (6), ionomycin (14), or monosodium urate (MSU) crystals (15), reportedly induce NETosis.

Currently, the functions of neutrophils are considered a double-edged sword: on one side, they exert pro-inflammatory actions during infections that contribute to the development of both innate and adaptive immunity (16, 17); on the other side, they are involved in tissue damage and in the initiation and perpetuation of immune dysregulation in chronic autoimmune diseases such as RA (18) and SLE (19). Nevertheless, neutrophils have also been implicated in the resolution of acute inflammation. When the initial wave of neutrophils is missing or when neutrophils are impaired in generating NETs, the inflammatory responses to zymosan or MSU tend to chronify (20, 21). In this case, an aggregate of NETs confines the inflammatory stimulus and degrades inflammatory cytokines and chemokines limiting further neutrophil recruitment and orchestrating the resolution of inflammation (20, 21).

We have recently shown that diamond nanoparticles induce membrane damage in a broad spectrum of cells *in vitro* and *in vivo* (22). The ability to penetrate through plasma membranes was also demonstrated for C60 fullerenes (23) and for single- and multi-walled carbon nanotubes (24). The exposure to carbon nanotubes of mice resulted in the formation of granulomata in skin, lungs, and peritoneum (25, 26). Taking together, metabolically inert nanoparticles induce sterile tissue damage upon injection. Since small nanodiamonds also cause NETosis in neutrophils (22), we hypothesized that such a necrosis-inducing trigger does not only mount sterile inflammation but also a robust resolution response. Thus, we aimed to create an *in vivo* model of permanent sterile inflammation to reveal the role of oxidative burst-dependent NETosis in the context of tissue damage in the absence of pathogens.

Here, we describe that nanodiamonds induced necrosis and self-limited sterile inflammation when injected into wild-type mice. Nanodiamonds also emerged as potent triggers for NETosis in cultured neutrophils. When nanodiamonds were injected into ROS-deficient mice lacking functional NETosis; however, the resolution of inflammation was severely impaired. NETs efficiently entrap nanodiamonds and shield healthy from necrotic tissue.

## Results

### Characterization of Diamond Particles

The structural and surface properties of the nanodiamonds, sized 10 nm, were verified by high-resolution transmission microscopy (HRTEM), X-ray photoelectron spectroscopy (XPS), and Raman spectroscopy. The Raman images of the diamonds are shown in Figure S1A in Supplementary Material. The spectra reveal the characteristic feature of diamonds with a peak at 1336/cm. The presence of graphitic carbon with bands at 1350 and 1580/cm is virtually absent. The XPS survey spectra of the diamonds showed signals of carbon (10 nm: 90.4%; 1000 nm: 90.1%) as dominant element together with the presence of oxygen (10 nm: 4.2%; 1000 nm: 9.6%) and nitrogen (10 nm: 1.8%; 1000 nm: 0.3%). Figure S1B in Supplementary Material shows (high resolution) transmission microscopy pictures of 10 nm nanodiamonds with a diameter of about 7 ± 4 nm. The lattice fringes with a spacing of 2.06 Å are assigned to the diamond (111) plane. The physicochemical properties of the diamonds are listed in Table 1.

**Table 1:** **Physicochemical characteristics of diamond particles**.

Particle size (nm)	Diameter/nm^a^	Hydrodynamic diameter/nm^b^ (pH = 7.4)	ζ-potential/mV (pH = 7.4)	Hydrophobicity
10	7 ± 4	618 ± 25	+34 ± 1	High
1000	1010 ± 300	970 ± 50	−38 ± 1	High

*^a^Determined by TEM*.

*^b^Determined by DLS*.

### Induction of Plasma Membrane Damage in Human Leukocytes

**Figure 1:**
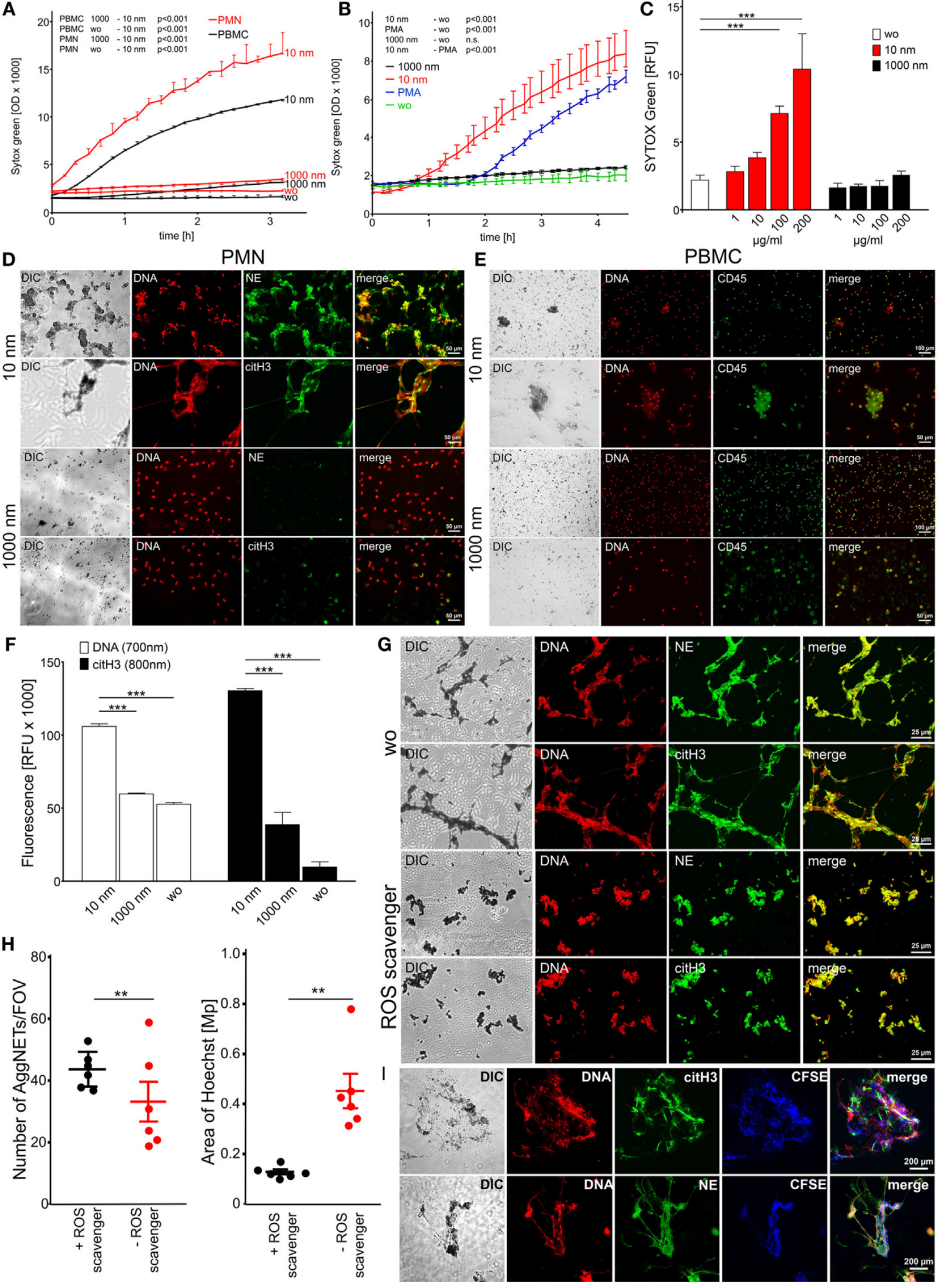
**Diamond nanoparticles induce plasma membrane damage and NET formation in human leukocytes**. **(A)** Assessment of DNA exposure in neutrophils (PMN) and peripheral blood mononuclear cells (PBMC). Cells without stimulus (wo) or incubated with nanodiamonds (10 nm) or microdiamonds (1000 nm) were measured using SYTOX Green. **(B)** Exposure of DNA by neutrophils was measured in response to phorbol 12-myristate 13-acetate (PMA) to nanodiamonds (10 nm) or microdiamonds (1000 nm) and without stimulus (wo) using SYTOX Green. **(C)** Dose-dependent increase of DNA exposure in PMN without stimulus (wo) or incubated with nanodiamonds (10 nm) or microdiamonds (1000 nm) by SYTOX Green after 150 min. **(D)** Microscopic analysis of PMN incubated with nanodiamonds (10 nm) or microdiamonds (1000 nm) and stained for DNA (Hoechst33342), neutrophil elastase (NE), or citrullinated histone H3 (citH3). Diamonds are visible in differential interference contrast (DIC) images. Cy5 fluorescence was artificially colored green. **(E)** Microscopic analysis of PBMC incubated with nanodiamonds (10 nm) or microdiamonds (1000 nm) and stained for DNA (Hoechst33342) and CD45-FITC. Diamonds are visible in DIC and provide strong background fluorescence in FITC. **(F)** Quantification of extracellular DNA and citH3 in human PMN without stimulus (wo) or incubated with nanodiamonds (10 nm) or microdiamonds (1000 nm) after 240 min. Significances below 0.001 are depicted. **(G)** Microscopic analysis of PMN incubated with nanodiamonds and treated with the ROS scavenger *N*-acetyl cysteine stained for DNA (Hoechst33342), NE, or citH3. Diamonds are visible in DIC. **(H)** *In silico* quantification of microscopic pictures regarding the number and area of DNA (Hoechst33342)-stained NET structures (AggNET) formed by PMN incubated with nanodiamonds in the absence or presence of the ROS scavenger *N*-acetyl cysteine. Each dot represents one analyzed field of view (FOV). **(I)** Microscopic analysis of NET formation in response to nanodiamonds induced necrosis of CFSE-labeled PBMC stained for DNA (Hoechst33342), NE, or citH3. Diamonds are visible in DIC. Data of one representative experiment reflecting the result of three independent experiments are shown as medians with interquartile ranges of triplicates. Two-way ANOVA **(A–C)**, one-way ANOVA **(F)**, Kruskal–Wallis one-way analysis of variance **(H)**, and Mann–Whitney *U* test **(I)** were used to evaluate differences among means; **p < 0.05, ***p < 0.001, and relative fluorescence units (RFU) field of view (FOV).

Freshly isolated human polymorphonuclear leukocytes (PMN) and peripheral blood mononuclear cells (PBMC) showed rapidly increasing membrane permeability, represented by an increase of the SYTOX Green signal due to its increased accessibility to DNA (Figure 1A) when exposed to 10 nm diamonds (nanodiamonds). SYTOX Green is a cell membrane-impermeable dye that specifically intercalates into accessible DNA, thereby increasing its fluorescence up to 500 times. Interestingly, the increase of the SYTOX signal in PBMC was less pronounced than the one observed in PMN. Next, we compared the SYTOX signal induced by nanodiamonds with the one induced by PMA as a classical stimulus for DNA externalization in conjunction with NETosis. In response to nanodiamonds, the SYTOX signal was enhanced earlier (60 min) and more pronounced than with PMA (Figure 1B). In contrast, upon incubation with the control 1000 nm diamonds (microdiamonds), the SYTOX signal was comparable to unstimulated cells, showing no membrane damage and no accessibility of the DNA in PBMC or PMN (Figure 1A). The membrane damage in response to nanodiamonds was dose dependent as represented by the increase of the SYTOX signal in PMN incubated with increasing amounts of particles (Figure 1C). We conclude that nanodiamonds induce rapid cell membrane rupture rendering DNA accessible in white blood cells.

In order to characterize the nuclear appearance of leukocytes upon contact with diamonds, we microscopically analyzed PMN and PBMC after co-incubation with nanodiamonds or microdiamonds. DNA was stained by propidium iodide or Hoechst 33342 and the DNA-associated proteins citH3 and NE by immunofluorescence. PMN incubated with nanodiamonds exhibited large spread aggregates composed of DNA and nanodiamonds co-localizing with citH3 and NE (Figure 1D). In contrast, employing microdiamonds no such structures were observed. The nuclei displayed a lobular shape, characteristic of neutrophils, and the signals of NE and citH3 were localized intracellularly. The nuclear appearance of PBMC incubated with nanodiamonds differed strongly from that of similarly treated PMN (Figure 1E). Only sporadic diamond and necrotic cell aggregates with normal nuclear morphology were observed in association with nanodiamonds. NET-like structures trapping nanodiamonds were not observed in PBMC samples. Incubation with microdiamonds induced nuclear modifications neither in PBMC nor in PMN (Figures 1D,E).

Live cell imaging confirmed the fast and uncontrolled rupture of the plasma membrane of PMN (Video S1 in Supplementary Material) and PBMC (Video S2 in Supplementary Material) in response to nanodiamonds. This process is represented by the conversion of the blue Hoechst3342 signal, being cell membrane permeable, to the red PI signal, intercalating into accessible DNA. The nuclear appearance of PMN markedly differed from that of PBMC. The lobulated nucleus of viable neutrophils, stained by Hoechst3342, became PI-positive, and displayed a decondensed morphology as soon as 30 min after the stimulus (Video S1 in Supplementary Material). This process was followed by externalization and spreading of the DNA. In contrast, nuclei of PBMC quickly became PI-positive, indicative for plasma membrane damage, but the DNA was not externalized and spread (Video S2 in Supplementary Material). Microdiamonds associated with both, PMN (Video S3 in Supplementary Material) and PBMC (Video S4 in Supplementary Material), but did not affect the integrity of their cellular membranes. However, some spontaneous cell death was observed most likely due to phototoxicity in time-lapse fluorescence microscopy (27).

Quantification of the signal for extracellular DNA and citH3 revealed a significant increase when PMN were incubated with nanodiamonds in comparison to microdiamonds or unstimulated cells (Figure 1F). Externalized chromatin of neutrophils co-localizing with citH3 is indicative for NETosis (11). In order to further evaluate NETosis, we added nanodiamonds to PMN pre-incubated with the ROS scavenger *N*-acetyl l-cysteine (NAC). Although we still observed NET formation in the presence of NAC, the aggregation of the nanodiamonds by NETs was reduced when compared to that resulting from the incubation of PMN with nanodiamonds alone (Figure 1G). This was confirmed by *in silico* morphometric quantification of NET aggregation concerning the number and area of NET structures (Figure 1H). In the presence of the ROS scavenger, the number of NET aggregates (AggNET) was significantly higher, but the area was significantly decreased. This reflects a reduced aggregation of NETs in the absence of ROS. Next, we employed CFSE-labeled PBMC, which had previously been incubated with nanodiamonds to stimulate freshly isolated viable PMN. Interestingly, we observed that necrotic PBMCs and nanodiamonds were entrapped in the NET aggregates characterized by large DNA filaments decorated with NE and citH3 (Figure 1I).

We conclude that nanodiamonds induced fast rupture of the plasma membrane when encountering leukocytes (PMN and PBMC). The mononuclear cells rapidly died by membrane rupture and became necrotic. Contrarily, PMN formed NETs, which tended to aggregate in the presence of ROS to confine nanodiamonds as well as necrotic mononuclear cells.

### Induction of Cellular Damage in Murine Bone Marrow-Derived Immune Cells

In order to determine the effect of nano- and microdiamonds on murine leukocytes, we quantified the accessibility of DNA for SYTOX Green in isolated bone marrow cells. To further analyze the role of the oxidative burst on nanoparticle-induced NETosis, experiments were conducted with bone marrow cells of both, WT and Ncf1** mice. Latter harbor a single-nucleotide polymorphism in the gene for the regulatory p47^phox^ subunit (*Ncf1*) of the NADPH oxidase NOX2. This mutation leads to strongly diminished NOX2-dependent ROS production resulting in a deficiency of neutrophils to undergo NOX2-dependent NETosis.

**Figure 2:**
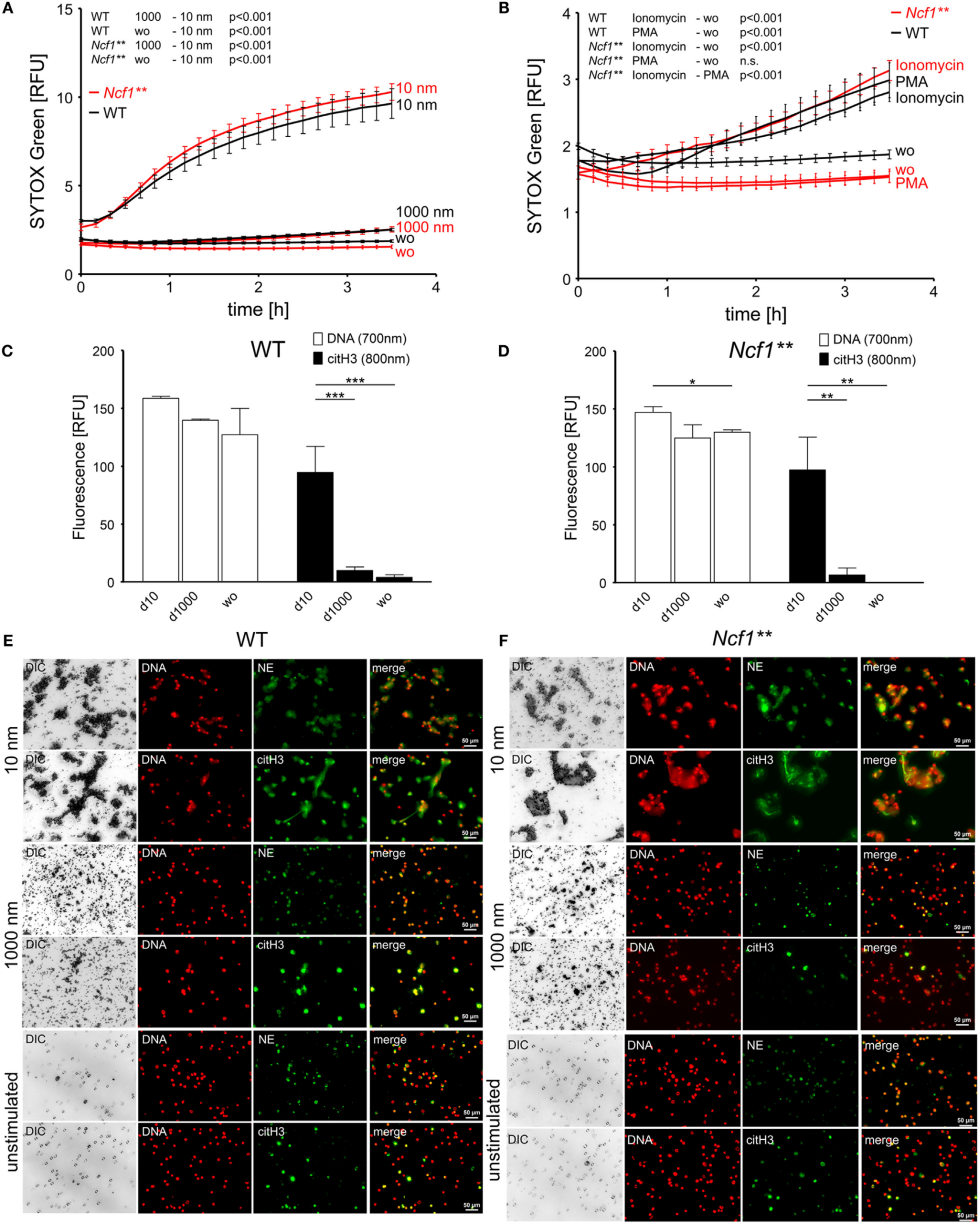
**Diamond nanoparticles induce plasma membrane damage and NET formation in murine bone marrow cells**. **(A)** Analysis of DNA exposure by bone marrow cells of WT and Ncf1** mice in response to nanodiamonds (10 nm) or microdiamonds (1000 nm) or without stimulus (wo) using SYTOX Green. **(B)** Analysis of DNA exposure by bone marrow cells of WT and Ncf1** mice in response to NET-inducing stimuli (PMA and ionomycin) or without stimulus (wo) using SYTOX Green. **(C)** Quantification of extracellular DNA and citrullinated histone H3 (citH3) in bone marrow cells of WT mice without stimulus (wo) or incubated with nanodiamonds (10 nm) or microdiamonds (1000 nm) after 240 min. **(D)** Quantification of extracellular DNA and citH3 in bone marrow cells of Ncf1** mice without stimulus (wo) or incubated with nanodiamonds (10 nm) or microdiamonds (1000 nm) after 240 min. **(E)** Microscopic analysis of bone marrow cells of WT mice incubated with nanodiamonds (10 nm) or microdiamonds (1000 nm) and unstimulated cells stained for DNA (Hoechst33342), neutrophil elastase (NE), or citH3. Diamonds are visible in differential interference contrast (DIC). Cy5 fluorescence was artificially colored green. **(F)** Microscopic analysis of bone marrow cells of Ncf1** mice incubated with of nanodiamonds (10 nm) or microdiamonds (1000 nm) and unstimulated cells stained for DNA (Hoechst33342), NE, or citH3. Diamonds are visible in DIC. Cy5 fluorescence was artificially colored green. Data of one representative experiment reflecting the result of three independent experiments are shown as medians with interquartile ranges of triplicates. Two-way ANOVA **(A,B)** and one-way ANOVA **(C,D)** were used to evaluate differences among means; *p < 0.05, **p < 0.01, ***p < 0.001, and relative fluorescence units (RFU).

Isolated bone marrow cells contained about 80% CD45^+^ leukocytes. More than 50% of these were identified as granulocytes (Figures S1C,D in Supplementary Material). Consistent with previous experiments employing human leukocytes, we observed a fast permeabilization of the plasma membrane in response to nanodiamonds in bone marrow cells of both, WT and Ncf1** mice (Figure 2A), while the microdiamonds were inert. Stimulation with ionomycin of bone marrow cells triggered DNA release in both, WT and Ncf1** mice (Figure 2B). This stimulus reportedly induces NETosis independent of NOX2-dependent ROS production (28). Importantly, only leukocytes from WT mice underwent DNA externalization in response to PMA, a NOX2-dependent ROS inducing stimulus (29), whereas Ncf1**-derived cells did not. Similar to human cells, the quantification of extracellular citH3 revealed significantly elevated signals after exposure to nanodiamonds in comparison to microdiamonds or unstimulated cells (Figures 2C,D). Analyses by microscopy of WT or Ncf1** bone marrow cells incubated with nanodiamonds revealed a similar appearance to that of human PMN (Figures 2E,F). The nanodiamonds induced NET structures of DNA co-localizing with citH3 or NE and sequestering the particles in both, WT- and Ncf1**-derived cells (Figures 2E,F). The microdiamonds did not induce the formation of such structures (Figures 2E,F). Lower magnification pictures showing NET structures or nuclear morphology in a larger area are depicted in Figures S1E,F in Supplementary Material. From these observations, we can conclude that the direct effects of nanodiamonds on both human and murine leukocytes are ROS independent.

### Tissue Damage-Induced Inflammation Does Not Resolve in Ncf1** Mice

**Figure 3:**
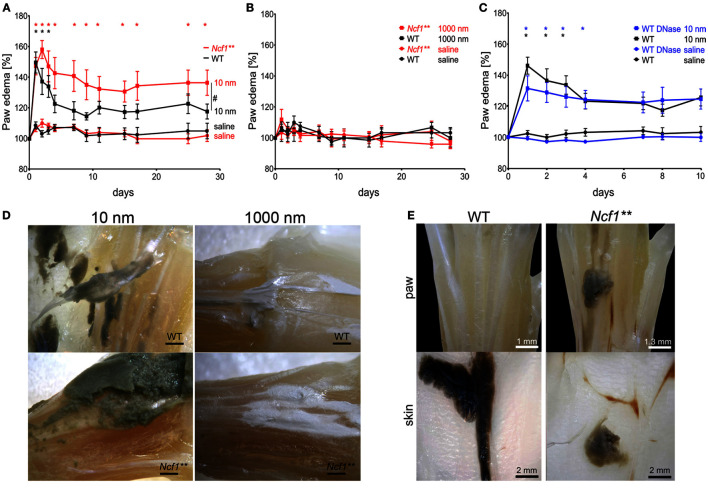

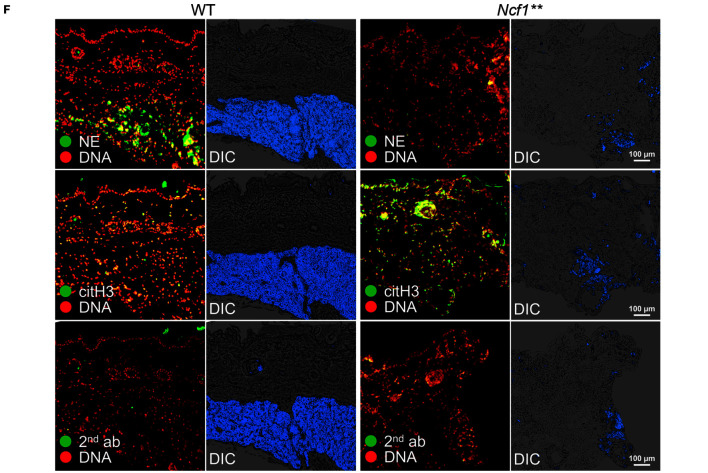
**Chronification of necrosis-associated sterile inflammation in NOX2-deficient mice**. **(A)** Paw edema in response to injected nanodiamonds in WT and Ncf1** mice. Means ± SEM of the relative hind paw thickness are shown. ^#^*p* < 0.05 of areas under the curves as determined by *t* test; *n* = 4–6. **(B)** Measurement of hind paw edema in response to injected microdiamonds in WT and Ncf1** mice. Means ± SEM of the relative hind paw thickness are shown. **(C)** Measurement of hind paw edema in response to injected nanodiamonds in WT mice in the absence or presence of DNase I. Means ± SEM of the relative hind paw thickness are shown. *p < 0.001; *n* = 5 **(D)** Paws of WT and Ncf1** mice microdissected 15 days after injection of nanodiamonds (dark gray matter, 10 nm) and of microdiamonds (white matter, 1000 nm). **(E)** Macroscopic pictures of dissected paws of WT and Ncf1** mice at day 28 after injection of nanodiamonds showing nanodiamonds (dark gray matter) on the paw and overlaying skin. **(F)** Paraffin sections of overlaying skins shown in **(E)**, stained for neutrophil elastase (NE), citrullinated histone H3 (citH3), and DNA (PI) as well as differential interference contrast (DIC). Nanodiamonds giving highest contrast in DIC were artificially colored blue. Data are shown as means ± SEM. Two-way ANOVA with Bonferroni post testing **(A–C)** was used to evaluate differences among means.

Nanodiamonds induced rapid and substantial cellular damage *in vitro* independently of the leukocyte type in both human and mice. Since nanodiamonds cannot be digested enzymatically, we hypothesized that their persistent presence might result in a continuous induction of inflammation *in vivo*. To investigate the role of NOX2-dependent NETosis in nanoparticle-induced inflammation, we injected 1 mg of nanodiamonds or microdiamonds into the metatarsal region of the hind paws of WT or Ncf1** mice. Paw edema in the particle-injected foot was recorded as a specific sign of local inflammation over 28 days and compared to the sham-treated control foot. Already 24 h after injection, we observed the development of significant paw edema in both mouse strains injected with nanodiamonds (Figure 3A). In WT mice, the inflammation resolved within 3 days. Ncf1** mice developed a sustained inflammation that did not resolve until the end of the experiment at day 28. In contrast, microdiamonds neither induced paw swelling in WT nor in Ncf1** mice (Figure 3B). In an additional experiment, we injected nanodiamonds in the presence of DNase I into WT mice (Figure 3C). To avoid further injection-induced tissue damage, DNase I was applied only once together with the nanodiamonds as well as i.v. 24 and 48 h after injection of nanodiamonds. Paw edema was recorded for 28 days. Since no differences between the groups at later time points were observed, values until day 10 are shown. DNase I-treated mice showed prolonged inflammation until day 5, while in the untreated group, the inflammation resolved already at day 4.

Dissection of the WT hind paws disclosed that nanodiamonds were wrapped in membrane-like structures resembling granulation tissue (Figure 3D). In contrast, Ncf1** hind paws showed bare nanodiamonds without visible association to connective tissue. The microdiamonds injected into hind paws showed no signs of clumping or granulation (Figure 3D). Moreover, nanodiamonds were tightly attached to the overlaying skin in WT paws, while they appeared more loose and spread in the surrounding tissues in the paws of Ncf1** mice (Figure 3E). Histological analysis of the skins revealed packing of nanodiamonds associated with DNA and NE in WT mice (Figure 3F). In contrast, nanodiamonds were dispersed in the skin section and not associated with extracellular neutrophil markers in Ncf1** mice.

In summary, nanodiamonds trigger a strong local inflammatory response when injected into tissues by inducing membrane rupture and necrosis. In WT mice, cell death in response to nanodiamonds induced ROS-dependent NETosis, organization of nanodiamonds in the tissue, and resolution of the initial inflammation. In the absence of NOX2-dependent NETosis; however, inflammation does not resolve, nanodiamonds are dispersed in the tissue, and this may trigger chronic inflammation.

## Discussion

Consistent with previous findings employing epithelial and red blood cells (RBC) (22), we report that nanodiamonds induce cellular damage in human and murine leukocytes *in vitro*. In contrast, microdiamonds did not affect the membrane integrity of these cells. In the case of PMN, rupture of the plasma membrane in response to nanodiamonds was accompanied by DNA decondensation and release of DNA decorated with citH3 and NE. These NETs sequestered and entrapped the nanodiamonds. NETosis induced by particulate matter has already been reported for MSU (30), calcium carbonate (31), calcium oxalate (32), and diamonds (22). The mechanism of crystal-induced NETosis is associated with the necroptosis pathway (33). Several other studies support a role for the size of nanoparticles and their effects on cells and tissues (34–36). Most of these studies are limited to macrophage-mediated particle engulfment (37, 38) and clearance (39). In the case of neutrophils exposed to nanomaterials, investigations focused on cytotoxicity (40–42), degranulation (43), or phagocytic uptake (44). Recently, MPO-mediated degradation of single-walled carbon nanotubes has been shown to abrogate carbon nanotube-induced pulmonary inflammation (45). Further reports on neutrophils reacting to tissue damage induced by nanoparticles are scarce. We have recently demonstrated that the size of particles critically determines NETosis, and this is associated with the resolution of an initial neutrophil-driven inflammation in air pouches (22). Microdiamonds of 1 μm were not taken up by granulocytes *in vitro*, whereas small nanodiamonds induced NETosis due to their size and hydrophobicity. This result seems to contradict other findings showing that neutrophils undergo NETosis selectively in response to large pathogens, but not in response to small yeast or single bacteria (46). However, it is likely that neutrophils respond differently to pathogens than nanodiamonds, which differ greatly in size and properties from pathogens like bacteria or yeast.

The role of NETosis in spatially and temporally restricted sterile inflammation following necrosis has not been investigated. Since nanodiamonds induced substantial cellular damage *in vitro* and persist at the site of injection, we hypothesized that they provoke chronic inflammation after *in vivo* injection. Indeed, we observed that nanodiamonds triggered inflammation within 24 h after injection. However, inflammation resolved within 3 days in WT mice despite continuous persistence of the nanodiamonds *in situ*. Conversely, mice deficient in NOX2 developed a sustained inflammatory response indicating a role for ROS-dependent NETosis in the resolution of necrosis-associated sterile inflammation. This observation is also supported by the fact that the formation of large NET aggregates is notably reduced in the presence of the ROS scavenger *N*-acetyl l-cysteine. Consistently, DNase I-treated mice showed prolonged paw edema due to NET degradation. However, the effect of DNAse I was not sufficient to induce chronification of nanodiamond-induced inflammation. Macroscopical analyses revealed that the diamonds were not enzymatically degraded, as expected, nor cleared from the tissue and remained at the site of injection. In contrast, we observed granulation-like tissue surrounding nanodiamonds exclusively in WT animals but not in ROS-deficient Ncf1** mice. Unfortunately, immunohistochemical evaluation of the whole paw could not be done, since diamonds precluded sectioning of the tissue samples. However, histological analyses of adjacent skin tissue collected at day 28 revealed tight packing of nanodiamonds in WT mice and anatomical association of nanodiamonds with neutrophil markers. Contrarily, Ncf1** mice lacking oxidative burst-depending NETosis were not able to encapsulate nanodiamonds and therefore the inflammatory trigger remained bare. These observations support the hypothesis that neutrophils contribute to the entrapping and isolation of particulate matter by NETosis.

Recently, we reported that the formation of aggregated NETs is implicated in the resolution of inflammation in patients with gout (20). NETs enclose MSU crystals and form tophi. This amorphous material can be clinically silent for a long time. Thus, the acute inflammation caused by MSU crystals is ameliorated if they are entrapped in large NET aggregates. This reaction represents the basis of granuloma formation in patients with gout (15). Similar mechanisms may operate during the infection with *Mycobacterium tuberculosis*, a condition characterized by massive granuloma formation (47). Thus, the formation of granuloma during an inflammatory process can be considered a mechanism to terminate excessive inflammation.

Necrotic injury is usually accompanied by massive neutrophil infiltration. Depending on the affected organ, it may result in acute life-threatening conditions (4). Neutrophil infiltration is the key initial process of the inflammatory response to sterile necrosis (48). We observed that the concurrence of necrotic cells and NETosis leads to the entrapment of the dead cells. A recent report on NETosis induced by apoptotic cells *in vitro* also supports the link between cell death and NETosis (49).

**Figure 4:**
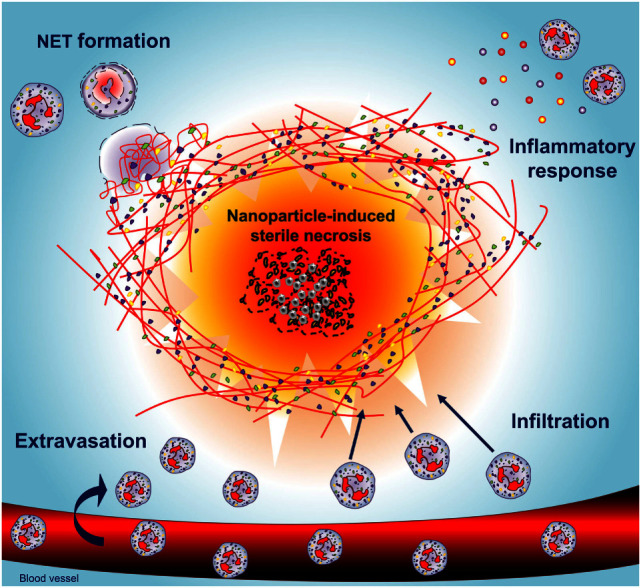
**Neutrophils shield necrotic tissue by the formation of NETs building an anti-inflammatory barrier**. Nanodiamonds induce necrosis by cell membrane damage, release of damage-associated molecular patterns, and the recruitment of neutrophils. Concerted NETosis of neutrophils then builds a barrier around the necrotic core consisting of aggregated NETs, which secludes the nanodiamonds and allows resolution of inflammation.

In summary, we conclude that aggregated NETs contribute to the resolution of sterile inflammation induced by nanoparticle-mediated cell necrosis. Neutrophils, recruited to sites of nanoparticle-induced cell death, undergo NETosis and form NET aggregates segregating the damaged area from the surrounding viable tissue (Figure 4). The initial inflammatory response may involve several cytotoxic mechanisms including ROS-independent NETosis. However, in the course of inflammation, the absence of ROS-dependent NETosis leads to a chronic inflammatory response. NET aggregates therefore essentially contribute to the termination of the inflammatory response.

## Materials and Methods

### Preparation of Human Material

All analyses of human material were performed in full agreement with institutional guidelines and with the approval of the Ethical committee of the University Hospital Erlangen (permit # 193 13B). Human peripheral PMN and PBMC were isolated from heparinized (20 U/ml) venous blood of normal healthy donors (NHD) by Lymphoflot (Bio-Rad, Hercules, CA, USA) density gradient centrifugation as described elsewhere (50). Briefly, whole blood was carefully pipetted on Lymphoflot solution and centrifuged for 30 min at 1400 rpm. Then, the plasma was carefully removed and the PBMC layer was collected. The PMN-rich layer on top of RBC was taken and subjected to hypotonic lysis of RBC. Cell viability was assessed by trypan blue exclusion.

### Mice

Ncf1** mice, harboring a single-nucleotide polymorphism in the gene for the regulatory p47phox subunit (Ncf1) of the NADPH oxidase NOX2 (51, 52), originate from The Jackson Laboratories and were backcrossed over more than 10 generations to the BALB/c background and maintained at the animal facilities of the University of Erlangen. The animal studies were approved by the Veterinary Office of the Government of lower Franconia (permit # 55.2 DMS-2532-2-103) and conducted according to the guidelines of the Federation of European Laboratory Animal Science Associations (FELASA). Genotyping of Ncf1** and WT littermates was done by pyrosequencing, as described (53).

### Characterization and Preparation of Diamond Particles

Transmission electron microscopy (TEM) images were recorded on a JEOL JEM-2011 electron microscope operated at an accelerating voltage of 200 kV. XPS measurements were performed with an ESCALAB 220 XL spectrometer from Vacuum Generators featuring a monochromatic Al Kα X-ray source (1486.6 eV) and a spherical energy analyzer operated in the CAE (constant analyzer energy) mode (CAE = 100 eV for survey spectra and CAE = 40 eV for high-resolution spectra), using the electromagnetic lens mode. No flood gun source was needed due to the conducting character of the substrates. The angle between the incident X-rays and the analyzer is 58°. The detection angle of the photoelectrons is 30°. Zeta (ζ) potential measurements were performed with a Zetasizer Nano ZS (Malvern Instruments S.A., Worcestershire, UK). The pH of all the samples was maintained at ~7.4. Micro-Raman spectroscopy measurements were performed on a Horiba Jobin Yvon LabRam HR micro-Raman system combined with a 473 nm (1 mW) laser diode as excitation source. Visible light is focused by a 100× objective. The scattered light is collected by the same objective in backscattering configuration, dispersed by a 1800-mm focal length monochromator and detected by a CCD camera. In order to exclude endotoxin contamination, diamonds were treated with NaOH, dried out in ethanol, and treated with 300°C heat before used in cultures and animal experiments.

### Nanoparticle-Induced Paw Swelling

We injected 1 mg of 10 or 1000 nm diamonds in 70 μl 0.9% sterile NaCl solution into the metatarsal region of the hind paws of Ncf1** and WT mice. The contralateral paw was injected with 70 μl 0.9% NaCl solution serving as control treatment. In order to assess the role of NETs in the resolution of nanoparticle-induced inflammation, we injected 1 mg of 10 nm diamonds in 70 μl 0.9% sterile NaCl solution containing 200 μg DNase I (Sigma-Aldrich) into the metatarsal region of the hind paws of WT mice. The contralateral paw was injected with 70 μl 0.9% NaCl solution containing 200 μg DNase I serving as control treatment. Additionally, 500 μg DNase I was injected intravenously 24 and 48 h after particle injection. To monitor paw edema as a sign of inflammation, foot pads were measured with an electronic caliper at the indicated time points. On day 28, mice were sacrificed by CO_2_ aspiration. Some mice were sacrificed at day 15 and hind paws were fixed in 4% paraformaldehyde then transferred to 70% ethanol for dissection.

### Isolation of Bone Marrow Cells

Bone marrows of femurs and tibiae of BALB/c WT and Ncf1** mice were flushed out with PBS and RBC were lysed using Tris-buffered (0.15 M) ammonium chloride (0.16 M) adjusted to pH 1.65.

### Plate Reader-Based Quantification of DNA Accessibility

Freshly isolated human PMN and PBMCs or murine bone marrow cells were adjusted to a concentration of 2 × 10^6^ cells/ml in HBSS containing 5.55 mM glucose, 1.2 mM calcium, and 0.5 mM magnesium (Thermo Fisher Scientific) and plated in 96-well plates (Greiner Bio-One, Frickenhausen, Germany) in a final cell density of 1 × 10^6^ cells/ml. The DNA dye SYTOX Green (Thermo Fisher Scientific) was added in a final concentration of 2.5 μM. Diamonds of 10 and 1000 nm were added in a final concentration of 200 μg/ml or as indicated. The traditional NETosis-inducing stimuli ionomycin (InvivoGen, San Diego, CA, USA) or PMA (Sigma, Darmstadt, Germany) were added in final concentrations of 1 μg/ml or 100 ng/ml, respectively. Plates containing PMA, ionomycin, 10 or 1000 nm diamonds in 100 μl of HBSS, respectively, were incubated at 37°C and 5% CO_2_ prior to addition of cells to equilibrate pH. Living cells were added and plates were covered. DNA externalization was analyzed for 210 min. on an Infinite^®^ 200 PRO plate reader (TECAN, Crailshaim, Germany) under controlled temperature. Excitation was performed at 485 nm and emission was detected at 535 nm. Values displayed in the graphs were normalized according to absorption or addition of fluorescence by 10 or 1000 nm diamonds, respectively.

### NETosis Assay for Subsequent Immunofluorescence Staining

For microscopic analysis, 200,000 per well freshly isolated PMN or PBMC or 600,000 murine bone marrow cells per well were seeded in RPMI (Thermo Fisher Scientific, Waltham, MA, USA) in LabTek2 Chamber Slides (Thermo Fisher Scientific, Waltham, MA, USA). After addition of 200 μg/ml, 10 or 1000 nm diamonds (Sigma-Aldrich, St. Louis, MO, USA), 10 μg/ml PMA (Sigma-Aldrich, St. Louis, MO, USA), or 1 μg/ml ionomycin (Sigma-Aldrich, St. Louis, MO, USA) cells were incubated for 4 h at 37°C and 5% CO_2_. For experiments using the ROS scavenger *N*-acetyl l-cysteine (NAC) (Sigma-Aldrich, St. Louis, MO, USA), PMN were pre-incubated for 30 min at 37°C and 5% CO_2_ with 5 mM NAC for 30 min before addition of 200 μg/ml 10 nm nanodiamonds and incubation for 4 h at 37°C and 5% CO_2_. For experiments with CFSE-labeled cells, freshly isolated PBMC were labeled with CellTrace™ CSFE (Thermo Fisher Scientific, Waltham, MA, USA) according to the manufacturer’s instructions. Then, cells were fixed with 0.1% paraformaldehyde (Sigma-Aldrich, St. Louis, MO, USA) for 15 min and stained, as described below.

### Histology and Immunofluorescence Staining

For analysis of murine tissue samples, histology was performed using paraffin embedding. Tissue samples were fixed overnight in 4% formalin, dehydrated with ethanol, and subsequently embedded in paraffin. This was followed by immunofluorescence staining. Chamber slides and deparaffinized sections were blocked for 18 h at 4°C with PBS containing 10% FBS (Merck Millipore, Billerica, Waltham, MA, USA). Primary antibodies detecting NE (ab21595, Abcam, Cambridge, UK) or citH3 (ab5103, Abcam, Cambridge, UK) were added to the slides in a 1:200 dilution and incubated for 1.5 h at room temperature. This was followed by incubation with the Cy5-conjugated secondary detection antibody AffiniPure Goat Anti-Rabbit IgG (H + L) (Jackson Immuno Research Labs, West Grove, PA, USA) in a dilution 1:400 for 1 h at room temperature in the dark together with Hoechst 33342 (Thermo Fisher Scientific, Waltham, MA, USA). Slides were washed with PBS and H_2_O and samples were embedded in DAKO fluorescent mounting medium (Agilent Technologies, Santa Clara, CA, USA). Slides were analyzed either using the Eclipse Ni-U (Nikon Corporation, Tokyo, Japan) or the BZ-X700 microscope (Keyence Corporation, Osaka, Japan). *Z*-stacks were performed to increase depth of field. Post-processing of pictures was performed using Photoshop CS5 (Adobe, München, Germany).

### Immunofluorescence Quantification of Samples Containing Diamond Particles

The 200,000 PMN or PBMC or murine bone marrow cells resuspended in RPMI were seeded in poly l-lysine (Sigma-Aldrich, St. Louis, MO, USA)-coated 96-well flat bottom cell culture plates. Cells were then incubated with 200 μg/ml 10 or 1000 nm diamonds, 10 μg/ml PMA (Sigma-Aldrich, St. Louis, MO, USA), or 1 μg/ml ionomycin (Sigma-Aldrich, St. Louis, MO, USA) for 4 h at 37°C, and 5% CO_2_. After incubation, DNA was stained with the membrane-impermeable DNA dye DRAQ7 (BioLegend, San Diego, CA, USA) at room temperature for 15 min. After centrifugation for 5 min at 1800 rpm, samples were measured using the near-infrared fluorescence imaging system Odyssey^®^ CLx Imaging System (LI-COR, Lincoln, NE, USA) at 700 nm. After DNA measurement, cells were fixed with 0.1% paraformaldehyde for 15 min and blocked for 18 h at 4°C in PBS containing 10% FBS. Samples were incubated with the primary antibody recognizing citH3 for 1.5 h at room temperature, followed by incubation with the secondary goat anti-rabbit detection antibody IRDye^®^ 800 CW (LI-COR, Lincoln, NE, USA) for 1 h at room temperature. After centrifugation for 5 min at 1800 rpm, samples were measured with the Odyssey^®^ Clx Imaging System at 800 nm. Post-processing of pictures and quantification of the signals was performed using Photoshop CS5 (Adobe, München, Germany).

### Live Cell Imaging

Isolated PMN and PBMC were adjusted to a concentration of 1 × 10^6^ cells/ml in RPMI. Cell suspensions were added to an 8-well Nunc chamber slide (VWR, Darmstadt, Germany). Chamber slides were pre-incubated at 37°C at least 30 min prior to addition of 200 μl of 10 or 1000 nm diamonds with a concentration of 200 μg/ml each. Staining solution containing 0.1 μg/ml Hoechst 33342 and 1 μg/ml PI in PBS was added shortly before addition of the diamonds. Slides were analyzed on a BZ-X710 microscope (Keyence, Neu-Isenburg, Germany). *Z*-stacks were performed to increase depth of field. Post-processing of pictures was performed with Photoshop CS5 (Adobe, München, Germany).

### Statistics

Results are represented as the mean ± SEM of the indicated number of replicates/experiments. We performed computations and created charts using the GraphPad Prism 5.03 software. For calculation of statistical differences among the groups, we used ANOVA test with Bonferroni *post hoc* correction, where applicable. Adjusted *p*-values <0.05 were considered to be statistically significant.

## Author Contributions

MB, MP, and JK planned and performed most of the *in vitro* and *in vivo* experiments, conducted data analysis, and wrote the manuscript. CR, DK, DW, CM, JH, and RoBi performed *in vivo* and *in vitro* experiments and conducted data analyses. AB, RaBo, and SS performed micro-Raman spectroscopy, X-ray photoelectron spectroscopy, and transmission electron microscopy analyses. GS and MaHo provided scientific input and wrote the manuscript. LM and MaHe supervised the project, planned and conducted experiments, data analysis, and wrote the manuscript. All the authors read and approved the manuscript.

## Conflict of Interest Statement

The authors declare that the research was conducted in the absence of any commercial or financial relationships that could be construed as a potential conflict of interest.

## Supplementary Material

Figure S1 **(A)** Raman spectra of used nanodiamonds. The spectra representing the features of 10 nm nanodiamonds (red) and 1000 nm microdiamonds (black) showing a characteristic peak at 1336/cm; **(B)** nanodiamonds are visualized using transmission electron microscopy (TEM) and high-resolution TEM (HRTEM). Lattice fringe spacing is depicted in angstrom **(A)** in the HRTEM picture. **(C)** Frequency of CD45^+^ cells in bone marrow preparations of WT (*n* = 2) and *Ncf1*** (*n* = 3) mice. Data are shown as means ± SEM. **(D)** Frequency of CD11b^+^ Ly6Cint cells in the population of CD45^+^ bone marrow cells. **(E)** Microscopic analysis of bone marrow cells of WT mice incubated with nanodiamonds (10 nm) or microdiamonds (1000 nm) stained for DNA (Hoechst33342), neutrophil elastase (NE), or citH3 at a low magnification. Diamonds are visible in differential interference contrast (DIC). Cy5 fluorescence was artificially colored green **(F)** Microscopic analysis of bone marrow cells of *Ncf1*** mice incubated with of nanodiamonds (10 nm) or microdiamonds (1000 nm) stained for DNA (Hoechst33342), NE, or citH3 at a low magnification. Diamonds are visible in DIC. Cy5 fluorescence was artificially colored green.

Video S1 **Time-lapsed fluorescence microscopy of polymorphonuclear leukocytes (PMN) and nanodiamonds**. Freshly isolated PMN (blue intracellular nuclei, Hoechst 33342) incubated with nanodiamonds undergo rapid rupture of the plasma membrane and subsequent release of DNA (red extracellular DNA, propidium iodide).

Video S2 **Time-lapsed fluorescence microscopy of peripheral blood mononuclear cells (PBMC) and nanodiamonds**. Freshly isolated PBMC incubated with nanodiamonds retain nuclear morphology (blue intracellular nuclei, Hoechst 33342) and undergo rapid rupture of the plasma membrane exposing DNA to the propidium iodide dye (red intracellular).

Video S3 **Time-lapsed fluorescence microscopy of polymorphonuclear leukocytes (PMN) and microdiamonds**. Freshly isolated PMN incubated with microdiamonds retain nuclear morphology (blue intracellular nuclei, Hoechst 33342) and do not release DNA to the extracellular space (propidium iodide).

Video S4 **Time-lapsed fluorescence microscopy of peripheral blood mononuclear cells (PBMC) and microdiamonds**. Freshly isolated PBMC incubated with microdiamonds retain nuclear morphology (blue intracellular nuclei, Hoechst 33342) and do not release DNA to the extracellular space (propidium iodide).
