# Corrigendum: Oxidative burst-dependent NETosis is implicated in the resolution of necrosis-associated sterile inflammation

**DOI:** 10.3389/fimmu.2025.1593749

**Published:** 2025-03-26

**Authors:** Mona H. C. Biermann, Malgorzata J. Podolska, Jasmin Knopf, Christiane Reinwald, Daniela Weidner, Christian Maueröder, Jonas Hahn, Deborah Kienhöfer, Alexandre Barras, Rabah Boukherroub, Sabine Szunerits, Rostyslav Bilyy, Markus Hoffmann, Yi Zhao, Georg Schett, Martin Herrmann, Luis E. Munoz

**Affiliations:** ^1^ Department of Internal Medicine 3 – Rheumatology and Immunology, Universitätsklinikum Erlangen, Friedrich-Alexander-University Erlangen-Nürnberg, Erlangen, Germany; ^2^ UMR CNRS 8520, Institut d’Electronique de Microélectronique et de Nanotechnologie (IEMN), Université Lille 1, Villeneuve d’Ascq, France; ^3^ Danylo Halytsky Lviv National Medical University, Lviv, Ukraine; ^4^ Department of Rheumatology and Immunology, West China Hospital, Sichuan University, Chengdu, China

**Keywords:** necrosis, inflammation, nanodiamonds, NETosis, resolution, reactive oxygen species

In the published article, there was an error in **Supplementary Figure S1B** as published. **Supplementary Figure S1B** of this Figure included by mistake a TEM image of a ND finally not used in this work.

The authors apologize for this error and state that this does not change the scientific conclusions of the article in any way. The original article has been updated.

