# Supplementary material for: Oxidative Burst-Dependent NETosis Is Implicated in the Resolution of Necrosis-Associated Sterile Inflammation
Source: Front Immunol. 2016 Dec 1;7:557. doi: 10.3389/fimmu.2016.00557 (PMC5131011; doi:10.3389/fimmu.2016.00557)
Supplement: Figure S1 — (A) Raman spectra of used nanodiamonds. The spectra representing the features of 10 nm nanodiamonds (red) and 1000 nm microdiamonds (black) showing a characteristic peak at 1336/cm; (B) nanodiamonds are visualized using transmission electron microscopy (TEM) and high-resolution TEM (HRTEM). Lattice fringe spacing is depicted in angstrom (A) in the HRTEM picture. (C) Frequency of CD45+ cells in bone marrow preparations of WT (n = 2) and Ncf1** (n = 3) mice. Data are shown as means ± SEM. (D) Frequency of CD11b+ Ly6Cint cells in the population of CD45+ bone marrow cells. (E) Microscopic analysis of bone marrow cells of WT mice incubated with nanodiamonds (10 nm) or microdiamonds (1000 nm) stained for DNA (Hoechst33342), neutrophil elastase (NE), or citH3 at a low magnification. Diamonds are visible in differential interference contrast (DIC). Cy5 fluorescence was artificially colored green (F) Microscopic analysis of bone marrow cells of Ncf1** mice incubated with of nanodiamonds (10 nm) or microdiamonds (1000 nm) stained for DNA (Hoechst33342), NE, or citH3 at a low magnification. Diamonds are visible in DIC. Cy5 fluorescence was artificially colored green. [file image_1.pdf]

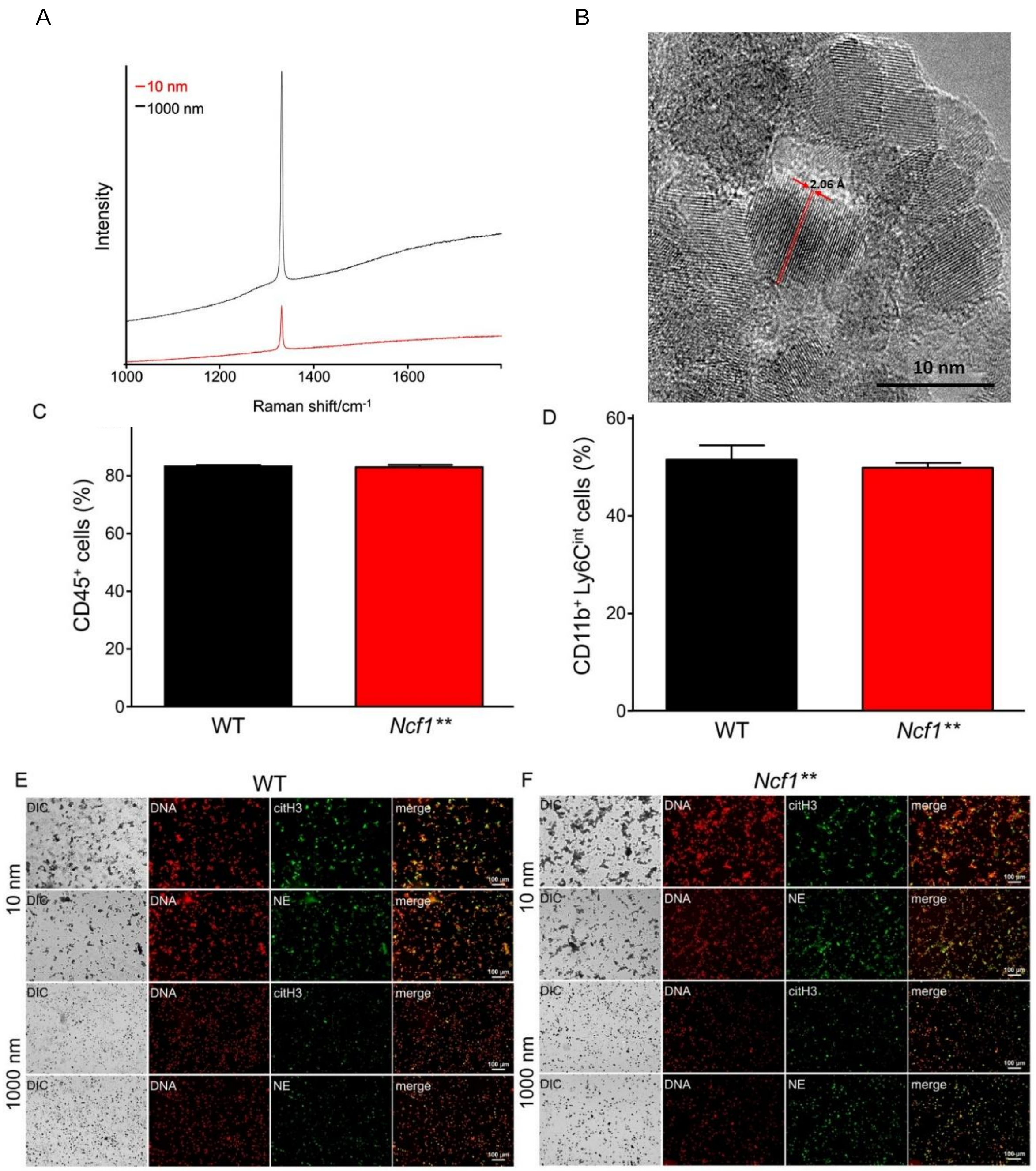

Figure S1. (A) Raman spectra of used nanodiamonds. The spectra representing the features of 10 nm nanodiamonds (red) and 1000 nm microdiamonds (black) showing a characteristic peak at 1336/cm; **(B)** nanodiamonds are visualized using high-resolution TEM (HRTEM). Lattice fringe spacing is depicted in angstrom in the HRTEM picture. **(C)** Frequency of CD45+ cells in bone marrow preparations of WT (n = 2) and Ncf1** (n = 3) mice. Data are shown as means ± SEM. **(D)** Frequency of CD11b+ Ly6Cint cells in the population of CD45+ bone marrow cells. **(E)** Microscopic analysis of bone marrow cells of WT mice incubated with nanodiamonds (10 nm) or microdiamonds (1000 nm) stained for DNA (Hoechst33342), neutrophil elastase (NE), or citH3 at a low magnification. Diamonds are visible in differential interference contrast (DIC). Cy5 fluorescence was artificially colored green **(F)** Microscopic analysis of bone marrow cells of Ncf1** mice incubated with of nanodiamonds (10 nm) or microdiamonds (1000 nm) stained for DNA (Hoechst33342), NE, or citH3 at a low magnification. Diamonds are visible in DIC. Cy5 fluorescence was artificially colored green.
